# Indoor air quality in remote first nations communities in Ontario, Canada

**DOI:** 10.1371/journal.pone.0294040

**Published:** 2023-11-22

**Authors:** Gary Mallach, Liu (Sunny) Sun, Michael McKay, Thomas Kovesi, Gail Lawlor, Ryan Kulka, J. David Miller

**Affiliations:** 1 Water and Air Quality Bureau, Health Canada, Ottawa, Canada; 2 Nishnawbe Aski Nation, Thunder Bay, Canada; 3 Department of Pediatrics, Children’s Hospital of Eastern Ontario (CHEO), University of Ottawa, Ottawa, Canada; 4 Energy Matters, Pickering, Canada; 5 Department of Chemistry, Carleton University, Ottawa, Canada; Satyawati College, University of Delhi, INDIA

## Abstract

A recent study of the health of Indigenous children in four First Nations Communities in remote northwestern Ontario found that 21% of children had been admitted to hospital for respiratory infections before age 2 years. Here we report a detailed analysis of the housing conditions in these communities. We employed a variety of statistical methods, including linear regression, mixed models, and logistic regression, to assess the correlations between housing conditions and loadings of biocontaminants (dust mite allergens, fungal glucan, and endotoxin) and indoor concentrations of PM_2.5_, CO_2_, benzene, and formaldehyde. The houses (n = 101) were crowded with an average of approximately 7 people. Approximately 27% of the homes had sustained CO_2_ concentrations above 1500 ppm. Most homes had more than one smoker. Commercial tobacco smoking and the use of non-electric heating (e.g., wood, oil) were associated with increased fine particle concentrations. Over 90% of the homes lacked working Heat Recovery Ventilators (HRVs), which was associated with increased fine particle concentrations and higher CO_2_. Of the 101 homes, 12 had mold damage sufficient to increase the relative risk of respiratory disease. This resulted from roof leaks, through walls or around the windows due to construction defects or lack of maintenance. A similar percentage had mold resulting from condensation on windows. Endotoxin loadings were much higher than any previous study in Canada. This work provides evidence for the need for more effort to repair existing houses and to ensure the HRVs are properly installed and maintained.

## 1. Introduction

Housing and housing conditions became an issue for the Government of Canada during World War II. Laws authorizing the national government to conduct research and support the construction of housing were passed during that period. Most Indigenous communities had very poor housing and petitioned the government to do more. In 1970, a program was developed to help improve housing in Indigenous communities to make them safer, warmer in winter and provide decent stoves. Two decades later, housing in Indigenous communities was still twice as likely as elsewhere in Canada to need major repairs [1]. Mold and dampness have long been a concern to First Nations communities in Canada [2, 3]. However, aside from mold and dampness, other factors such as the use of older wood stoves as a primary heating source and inadequate ventilation contribute to reduced indoor environmental quality (IEQ) in housing. In support of several indoor air quality guidelines [4–6], various groups including the American Industrial Hygiene Association and the American Academy of Allergy Asthma & Immunology developed guidelines on inspecting and addressing IEQ problems in homes [7–9]. These guidelines provide a framework for properly addressing the issues.

Over the past forty years, comprehensive studies of IEQ in residential housing have been done in communities representing climates across Canada including over 1,200 homes in Vancouver, Edmonton, Wallaceburg, Windsor, Toronto, Nunavut, Ottawa, Prince Edward Island (PEI), and Halifax [10–16]. Outside Nunavut, these studies revealed that the percentage of homes with dampness problems has stubbornly remained at ~20% [16]. A recent analysis indicated that a similar percentage of homes in the USA have mold and dampness problems [17]. In health studies, negative respiratory outcomes are associated with visible mold and dampness above a threshold [15, 18]. Typically, >1% of visible mold and dampness per unit floor area is required to elicit a health effect on a population level [19].

Previous studies have identified factors leading to mold and dampness in residential housing. A study in Wallaceburg in humid southern Ontario demonstrated that the single largest cause of mold and moisture was condensation from the wicking of water into basements [20]. The range of visible mold damage was 0.04–3.0 m^2^. In homes in PEI, inadequate ventilation resulted in visible mold in 59–71% of windows and bathrooms, 18% of below-grade walls, along with evidence of water in the attic in an additional 12%. The range of visible mold was 0.01–6.5 m^2^ [21]. In Ottawa, mold was more common in rural areas than urban dwellers, with visible mold present in half the rooms above grade level and the basements [22]. The range of visible mold was similar to that found in PEI and Wallaceburg.

Overall, approximately 16% of First Nations housing in Canada are reported to need major repairs and 17% are over-crowded (more than one person per room) [23]. However, these rates vary widely, related to remoteness, rurality, and community income levels [24, 25]. Few comprehensive studies have conducted in First Nations Communities. One such study was in the Elsipogtog Reserve, New Brunswick. In this community, visible mold was observed in ~70% of the homes, and 15% had visible mold greater than 1–2% including one with very serious mold damage. Most of the mold damage was due to basement leaks from improper drainage and waterproofing as well as roof leaks in the communities discussed above. Mold around window frames due to condensation was also common. Overall, the nature of the problems was largely similar to those in previous studies in Canada but in some houses, the damage was much greater. The more serious mold damage in Elsipogtog tended to be due to causes that could progress to hazardous conditions [26].

We recently completed a study of child health and housing in four remote First Nations communities in northwestern Ontario. One-fifth of the children in the study had been admitted to the hospital for respiratory infections before the age of two [24]. The purpose of this paper is to describe in more detail the housing conditions and IEQ in these four First Nations communities and to identify potential influential factors.

## 2. Materials and methods

### 2.1 Study design

As noted, this study was conducted in four First Nations communities in Northwestern Ontario, three of which were not accessible by road except during a brief period in winter [24]. This is in an area known to have high rates of children requiring treatment and/or hospitalization for respiratory disease [27]. These communities are predominantly Anishinaabe with some Cree communities and are among the more than 30 remote communities for which Meno Ya Win Health Centre in Sioux Lookout, Ontario serves as the regional hospital.

Research Ethics Board approvals were obtained from Health Canada, the Children’s Hospital of Eastern Ontario (CHEO), the Ottawa Hospital, and the Sioux Lookout Meno Ya Win Health Centre. This project was developed under the guidance of the Nishnawbe Aski Nation and the Sioux Lookout First Nations Health Authority. Permission was granted and support was provided by each of the four communities. Written consent was obtained from a member of each household prior to data collection. The Nishnawbe Aski Nation has full ownership of the resulting data [24].

### 2.2 Data collection

Houses were selected as described in Kovesi et al. 2022 [24]. Briefly this comprised First Nations families with children 3 years old or less. We recruited a substantial proportion of eligible homes (40%) in these small communities (~1200 residents each). Samples were taken and the homes were inspected by an indoor air quality specialist. Protocols were adapted from previous research by Health Canada and Canada Mortgage & Housing Corporation and the American Industrial Hygiene Association [7, 9, 21]. The surface area of visible mold and moisture damage was measured and recorded along with the cause(s). Baseline information was collected through inspection and questionnaire (e.g., Heat Recovery Ventilator (HRV) condition and use; primary and secondary heating; wood stoves, exhaust ventilation; indoor wood storage, commercial tobacco smoking).

Settled dust samples were collected from the living room floor. The floor samples were collected using an x-cell 100 dust sock fitted to the hose of an Omega HEPA vacuum cleaner (Midwest Filtration Company, Cincinnati, OH, USA) with technicians aiming to collect at least 2 g of dust per sample, typically from over 1–2 m^2^ of flooring. Sampling time was approximately 4 minutes per collection. If insufficient material was collected in this time, additional time was taken to vacuum a larger area. Surface type (e.g., carpet, hard surface) and size of the area vacuumed were logged, to calculate biocontaminant loading per floor surface area sampled. This sampling protocol has been demonstrated to be quantitative for allergens [26]. Samples were transported and stored under air dry conditions and the dust was sieved to 300 micrometers and weighed. The dust <300 micrometers was analyzed for endotoxin, house dust mite allergens, and 1,3-beta-D-glucan. Endotoxin was analyzed by the Limulus Amoebocyte Lysate assay according to the manufacturer’s instructions (Associates of Cape Cod, Falmouth, MA, USA). Fungal glucan was analyzed by the Factor G based LAL assay [28]. The dust-mite allergens Der f 1 (*Dermataphagoides farinae*) and Der p 1 (*Dermataphagoides pteronyssimus*) were extracted with borate buffer and determined with monoclonal-based enzyme immunoassays (Indoor Biotechnologies #EL-DF1, #EL-DP1; Charlottesville, VA, USA). Glucan and endotoxin load were calculated by dividing their weights by the surface area of flooring vacuumed [29, 30].

Continuous indoor PM_2.5_ (5-min integration) was measured in each home using DustTrak 8530 (TSI, St. Paul, MN, USA). The DustTrak measurements were found to over-estimate PM_2.5_ concentrations by a factor of 4, compared to collocated gravimetric measurements. Continuous indoor CO_2_ was measured using a Vaisala GMW93 CO_2_ Sensor (Vantaa, Finland) attached to a HOBO data logger (Onset, Cape Cod, MA, USA). A separate HOBO data logger was used to collect indoor temperature and relative humidity data. These data were each collected for approximately 5 days. All data was collected between November, 2017, and March, 2019, during the coolest months when the homes are more tightly sealed.

### 2.3 Statistical analysis

The biocontaminant data (dust mite allergens, fungal glucan, and endotoxin) were expressed as loadings (per square meter of the area sampled). To estimate the below detection data (if less than 80% of samples), we used robust regression on order statistics (ROS) [16]. The loadings of biocontaminant data, and concentrations of PM_2.5_, CO_2_, benzene, and formaldehyde followed a lognormal distribution, and therefore, the descriptive statistics were presented using geometric mean (GM), geometric coefficient of variation (GCV), and 95% confidence interval (CI). We also reported the arithmetic mean (AM) and interquartile range (IQR) to facilitate comparison with other studies.

To identify influential factors affecting IEQ in First Nations homes, we considered a range of factors for analysis. These include general housing characteristics, such as dwelling type, location, house size, main and secondary heating fuel, hot water fuel, cooking fuel, presence of gas or propane appliances, ventilation systems, room or central air conditioning, window and door conditions, weather stripping condition, and floor material. Occupant demographics were considered including, the number of home occupants and the number of commercial tobacco smokers in the household. Additionally, quantitative information on the area of visible mold and dampness at all locations in the house, basement and/or crawlspace, including the presence of visual mold, mold odor, and current or past (within the last 12 months) wetting events was included. Finally, we examined other direct or indirect sources/factors, such as the general upkeep of the home, the type of vacuum cleaners, the presence of humidifiers and dehumidifiers, interior wood storage, presence of pets and pests.

To analyze the biocontaminant data and daily mean PM_2.5_ and CO_2_ concentrations, we first log-transformed the data. We used simple linear regression to investigate how individual factors affected loadings of fungal glucan and endotoxin, as well as concentrations of benzene and formaldehyde. For daily mean PM_2.5_ and CO_2_ concentrations, we used a simple linear mixed model with a first-order autoregressive covariance structure to identify significant factors. We included random intercepts in the models to account for correlations between repeated measures from each home. We calculated the exponentiated predictor parameter estimates and 95% CI to estimate the percent change in exposure associated with per unit change of continuous variable or the positive category of the dichotomous variable. We dichotomized surface mold as above or below 0.2 m^2^, based on previous data relating this threshold to wheezing illness in infants [31]. For the presence of mold odor and presence of visible mold area exceeding 0.2 m^2^, we used logistic regression to determine the effect of individual influential factors. The effect on odds, quantified as percent change (100 × (OR-1)), and probabilities (100 × OR / (1 + OR)) along with their corresponding 95%CI are presented. All the analyses were conducted using SAS Enterprise Guide 7.1 (SAS Institute Inc.). All First Nations homes inspected and monitored for IEQ were included in our analyses. No personally identifying information relating to study participants was available to the authors, as data only contained coded and de-identified home IDs.

## 3. Results

### 3.1 Household characteristics

One hundred and one residences were included in the analysis. This included all First Nations homes inspected and monitored, whereas the results in our previous article excluded homes of newborns and where children had recently moved in. Therefore, our results reflect the full dataset, a larger sample size than that of the previous publication [24]. The houses were predominately detached bungalows with a median home size of less than 100 m^2^ (median = 83m^2^, range 43-168m^2^). The houses were mainly constructed of 2”x 6” wood frames, with a 4/12 roof pitch. The layout was typically open concept for the kitchen and living/dining room with bedrooms off a hallway and one bathroom. Most were crowded, with a mean occupancy of 6.6 (SD = 2.6, range 3–17) people per house [24]. The most common fuel type for heating was wood (48%), followed by mixed (30%), electricity (16%), and oil (7%). Heat distribution was mainly by local convection. Only 10% of the wood stoves were EPA certified for lower emissions (80 FR 13671 2015). Commercial tobacco smoking was present in 94% of the houses. Where present, the mean number of commercial tobacco smokers per house was 2.6 and ranged up to 7. Although 44% of houses had an HRV, only 8% were in good working condition. A general description of the homes is presented in Table 1.

**Table 1:** General description of the sampling homes (N = 101).

Household characteristic		N (%)
Sampling community	Big Trout Lake	23 (23%)
	Kasabonika	24 (24%)
	Lac Seul	22 (22%)
	Sandy Lake	32 (32%)
Dwelling type	Detached house	90 (89%)
	Townhouse, semi-detached, or plex	8 (8%)
	Apartment	2 (2%)
	Mobile unit	1 (1%)
Home size (m^2^)	<100	81 (80%)
	100–150	18 (18%)
	≥150	2 (2%)
Number of residents	1–5	37 (37%)
	5–10	53 (52%)
	≥ 10	11 (11%)
Presence of smokers	Yes	95 (94%)
	No	6 (6%)
Fuel for heating	Electricity	16 (16%)
	Oil	7 (7%)
	Wood	48 (48%)
	Mixed	30 (30%)
Fuel for cooking	Electricity	97 (96%)
	Oil	3 (3%)
Presence of range hood	Yes	54 (53%)
	No	47 (47%)
Whether the range hood was vented to the outside	yes	53 (52%)
	No	47 (47%)
HRV in regular use, and in good working order	Yes	8 (8%)
	No	93 (92%)
Use of humidifier	Yes	46 (46%)
	No	55 (54%)
Use of dehumidifier	Yes	1 (1%)
	No	100 (99%)
Presence of visible mold	Yes	46 (46%)
	No	55 (54%)
Visible mold area exceeding 0.2 m^2^	Yes	19 (19%)
	No	82 (81%)
Presence of mold odor	Yes	23 (23%)
	No	78 (77%)
If any mold-specific cleaning has been done	Often	41 (41%)
	Rarely	29 (29%)
	Never	31 (31%)
Presence of water damage events in the past 12 months	Yes	62 (61%)
	No	39 (39%)
Presence of carpet	Yes	11 (11%)
	No	90 (89%)
Presence of pets	Yes	22 (22%)
	No	79 (78%)
Presence of pests	Yes	41 (41%)
	No	60 (59%)

Almost half of the homes had visible mold (46%), and 23% had mold odor. Of these, 12 had >1% visible mold and moisture damage per unit floor area, some much greater. Mold-specific cleaning was done often in 41% of homes, rarely in 29%, and never in 31%. More than half of the homes had experienced water damage events in the past 12 months (61%). Approximately 13% of the homes with crawl spaces had serious moisture damage and/or were improperly constructed.

Overall, many of these houses had serious problems with the building or its systems. The HRV air intake for two of these homes was located too close to the furnace exhaust. About 10% of the units had material mold damage due to leaks in the building fabric (walls, around windows, roof). A similar percentage had mold resulting from condensation on windows. Plumbing leaks, mainly in the bathrooms, were present in ~20% of the homes. More detail on the principal moisture failures is available in Kovesi et al. (2022) [24]. Other problems included a lack of air sealing around windows; roof leaks leading to water infiltration and subsequent mold growth; and improper stove connections. Most of the homes needed minor repairs and approximately half needed major repairs—some urgent.

### 3.2 Pollutant concentration and determinants

The average weight of dust (<300 μm) collected in the houses was 0.80 g (SD = 1.20) and the average loading was 0.60 g/m^2^ (SD = 1.12). Table 2 shows the range and variation of biocontaminant loadings, as well as PM_2.5_ and CO_2_ concentrations. Der p 1 was more prevalent than Der f 1, for which the samples were below the detection limit for most homes (82%).

**Table 2:** Geometric mean (GM), geometric coefficient of variation (GCV), 95% confidence intervals (CI) of GM, arithmetic mean (AM), and interquartile range (IQR) of biocontaminant loadings and air pollutant concentrations.

Pollutant	N (homes)	% BDL^†^	GM (GCV), 95%CI of GM	AM (IQR)
Der f 1 (ng/m^2^)	86	82%	1.1 (3.8), 0.73–1.5	4 (0.30–4.0)
Der p 1 (ng/m^2^)	89	28%	8.6 (11), 5.4–14	176 (2.0–24)
Endotoxin (EU/m^2^)	93	0%	42368 (9.1), 27453–65387	542724 (12796–136712)
Fungal glucan (μg/m^2^)	93	0%	48 (5.7), 33–71	264 (14–190)
Benzene (μg/m^3^)	97	24%	1.7 (0.87), 1.46–1.98	2.2 (0.98–2.93)
Formaldehyde (μg/m^3^)	99	0%	16. (0.90), 14–19	20 (11–27)
PM_2.5_ (μg/m^3^)	93	-	35 (1.8), 32–38	68 (17–69)
CO_2_ (ppm)	99	-	1053 (0.4), 1021–1086	1140 (818–1314)

^**†**^ BDL: below the detection limit.

Our results for Der p 1 and Der f 1 rely on the newer assay standard for measuring mite allergen concentrations, so to compare our results to earlier studies (pre-2012), we applied a correction factor to the results from the earlier Canadian studies (dividing by 1.7 for Der p 1 and by 12.7 for Der f 1) [32]. A summary of converted results of these studies is presented in S1 Table. Comparing the mite allergen loadings to findings from other Canadian studies, we observed that the Der p 1 GM loading in First Nations homes was higher than homes in Edmonton, Montreal, and Windsor, but lower than observed in Halifax, Toronto, and Ottawa [12, 16, 22]. The Der f 1 GM loading in this study of First Nations homes was lower than other Canadian cities such as Halifax, Windsor, Toronto, and Ottawa, but slightly higher than Edmonton and Montreal [12, 16, 22].

The GM of indoor PM_2.5_ was consistent with the range reported by Health Canada for homes with commercial tobacco smokers (less than 35 μg/m^3^) [6]. The GM of CO_2_ was slightly above the long-term exposure limit of 1000 ppm recommended by Health Canada [4]. These fell into three categories. The majority of the homes had CO_2_ values with occasional excursions over 1000 ppm. Some 19.4% of homes had sustained periods greater than 1500 ppm. Finally, 7% of the homes with sustained CO_2_ values above 2000 ppm. Of the latter, three were also moldy. The average temperature and relative humidity in the homes were 25.6°C (SD = 2.7) and 28.2% (SD = 7.8), respectively.

The factors affecting the loadings of biocontaminants, and concentrations of PM_2.5_ and CO_2_ in the First Nations homes are shown in Table 3. Higher levels of endotoxin were associated with the presence of carpets and pets in the home and tended to be associated with more firewood stored in the living space. Glucan was positively associated with the presence of carpets, firewood storage, a dirt floor crawlspace, and negatively with central forced air heating and continuous heating. Commercial tobacco smoking in the house, the use of woodstove, fireplace, or burning wood indoors, oil heating, and the use of stovetop or oven for cooking were positively associated with PM_2.5_ levels. Electric heating, the heating distribution system being central forced air, frequent floor cleaning, use of a vacuum cleaner, and HRV use were associated with lower PM_2.5_ levels. Unsurprisingly, CO_2_ values were positively associated with both the number of people living in the house, as well as mold and humidity. Benzene and formaldehyde were positively associated with commercial tobacco smoking, the use of stovetop or oven for cooking, as well as the woodstove, fireplace, or burning wood indoors, and negatively associated with indoor plants, operating a functioning HRV, and in the case of benzene concentrations, continuous ventilation. Possibly due to back drafting from the woodstove, formaldehyde was positively associated with having a range hood vented outdoors.

**Table 3:** Percent change in air pollutant concentrations by household characteristics with 95% confidence intervals (CI). Results for endotoxin, fungal glucan, benzene, and formaldehyde were determined by simple linear regression, and results for PM_2.5_ and CO_2_ were determined by simple linear mixed regression.

Pollutant	Household characteristic	Percent change (%) (95%CI) ^†^
Endotoxin (EU/m^2^)	If there are carpets in the living room, child’s bedroom, or mother’s bedroom (yes)	1642 (418, 5760) ***
	Are there any pets at home (yes)	236 (24, 810) *
	Total amount of firewood stored in child’s bedroom, living space, and kitchen (m^3^)	362 (-8, 2210)
Fungal glucan (μg/m^2^)	If the vacuum surface is carpet (yes)	1130 (389, 2993) ***
	If there are carpets in the living room, child’s bedroom, or mother’s bedroom (yes)	1821 (568, 5426) ***
	Total amount of firewood stored in child’s bedroom, living space, and kitchen (m^3^)	496 (45, 2347) *
	If ventilation is continuous (yes)	-59 (-81, -11) *
	Are there any pets at home (yes)	168 (10, 554) *
	If the main heating distribution system is central forced air (yes)	-67 (-88, -7) *
	Dirt floor crawl space (yes)	210 (5, 818) *
PM_2.5_ (μg/m^3^)	If the main heating system fuel is electric (yes)	-57 (-73, -30) **
	If the main heating distribution system is central forced air (yes)	-55 (-74, -24) **
	Average number of cigarettes/pipes smoked in house/day since last visit (per additional cigarette smoked)	145 (34, 349) **
	Are there any use of woodstove, fireplace, wood burned in house since last visit (yes)	61 (9, 138) *
	Are there any use of stovetop/oven since last visit (yes)	60 (6,140) *
	Number of times the stovetop/oven has been used since the last visit	2 (0, 4)
	How many times per week is living room floor swept or vacuumed (per additional floor cleaning)	-3 (-6, 0) *
	Number of smokers at home	15 (1, 30) *
	If using a vacuum cleaner (yes)	-48 (-72, -4) *
	If HRV in regular use, and in good working order (y/n)	-49 (-75, 3)
CO_2_ (ppm)	Living room mean relative humidity (%)	3 (2, 4) ***
	Number people usually live in this house	6 (3, 8) ***
	If the below grade wall is finished (yes)	41 (7, 84) *
	Number of smokers at home	6 (1, 12) *
	If the condition of outside wall is good, respect to water penetration (yes)	18 (2, 36) *
	If mold specific cleaning has been done (yes)	27 (6, 53) *
	If there are any crawl space vents (yes)	-14 (-25, 0)
	Are there any mold odors (yes)	18 (0, 40)
	If there is visible mold presented (yes)	14 (-1, 32)
Benzene (μg/m^3^)	Are there any use of stovetop/oven since last visit (yes)	70 (18, 145) **
	Number of times the stovetop/oven has been used since the last visit	2 (0, 4) *
	Are there any use of woodstove, fireplace, wood burnt in house since last visit (yes)	72 (27, 132) ***
	If the main heating system fuel is electric (yes)	-48 (-65, -23) **
	If ventilation is continuous (yes)	-36 (-53, -14) **
	Number of smokers at home	17 (5, 29) **
	If the main heating system fuel is wood (yes)	53 (14, 105) **
	If HRV in regular use, and in good working order (yes)	-47 (-69, -10) *
	If there are indoor plants (yes)	-42 (-64, -5) *
	Chimney height from lowest floor (m)	7 (0, 16)
Formaldehyde (μg/m^3^)	Are there any use of stovetop/oven since last visit (yes)	170 (95, 275) ***
	Number of times the stovetop/oven has been used since the last visit	4 (3, 6) ***
	Are there any use of woodstove, fireplace, wood burnt in house since last visit (yes)	67 (23, 128) **
	Is range hood vented outdoors (yes)	80 (35, 140) ***
	If there are indoor plants (yes)	-50 (-69, -18) ***
	If the range hood is presented and used (yes)	49 (10, 100) ***
	If HRV in regular use, and in good working order (yes)	-46 (-69, -6) *
	Number of smokers at home	10 (-1, 23)

^**†**^ Significant at the levels

*p < .05

** p < .01

*** p < .001

Mold odor was positively associated with visible mold area, wetting events, CO_2_ and relative humidity, and more likely when window and outside wall conditions were poor, with respect to water penetration (Table 4). Visible mold >0.2m^2^ was positively associated with wetting events, water penetration of the outside walls, wooden or damaged window frames, and visible mold below grade; it was negatively associated with the use of a range hood and bathroom fans, and the use of continuous ventilation.

**Table 4:** Percent change in odds and probability of presence of mold odor and visible mold area exceeding 0.2 square meters by household characteristics, with 95% confidence intervals (CI). Results were determined by logistic regression analysis.

Pollutant	Household characteristic	Percent change (%) (95%CI)^†^	Probability (%) (95%CI)^†^
Mold odor	If there is any visible mold growth below grade (yes)	4123 (391, 36190) **	98 (83, 100) **
	If there are any wetting events in the past 12 months (yes)	471 (57,1981) **	85 (61, 95) **
	Mean CO_2_ concentration (100 ppm)	12 (1, 23) *	53 (50, 55) *
	If the condition of window is good, respect to water penetration, outside view (yes)	-67 (-87, -12) *	25 (11, 47) *
	Total area of visible mold on the above and below grade floors (m^2^)	137 (8, 421) *	70 (52, 84) *
	Living room mean relative humidity (%)	7 (0.4, 14) *	52 (50, 53) *
	If the condition of outside wall is good, respect to water penetration (yes)	-60 (-85, 4)	29 (13, 51)
	If there are any crawl space vents (yes)	135 (-9, 510)	70 (48, 86)
	If the weatherstripping is in good condition (yes)	-61 (-87, 16)	28 (12, 54)
	If there is visible mold presented (yes)	124 (-14, 479)	69 (46, 85)
Mold area > 0.2 m^2^	If the range hood is presented and used (y/n)	-82 (-95, -41) **	15 (5, 37) **
	If the condition of outside wall is good, respect to water penetration (yes)	-79 (-93, -37) **	17 (6, 39) **
	Are there any mold odors (yes)	337 (50, 1173) **	81 (60, 93) **
	If the condition of window is good, respect to water penetration, outside view (yes)	-78 (-93, -34) **	18 (7, 40) **
	If there are any wetting events in the past 12 months (yes)	1455 (98, 12096) **	94 (66, 99) **
	If there is any visible mold growth below grade (yes)	540 (57, 2515) *	86 (61, 96) *
	If ventilation is continuous (yes)	-78 (-94, -20) *	18 (6, 45) *
	Are there any window damages (yes)	241 (12, 933) *	77 (53, 91) *
	If window frames are wooden (yes)	650 (16, 4756) *	88 (54, 98) *
	Functioning bathroom fan in every bathroom, used by occupant (yes)	-67 (-89, -3) *	25 (10, 49) *
	Mean CO_2_ concentration (100 ppm)	10 (-0.1, 20)	52 (50, 55)
	Building services sealed (yes)	-62 (-87, 10)	28 (12, 52)
	Living room mean relative humidity (%)	6 (-0.7, 13)	51 (50, 53)

^**†**^ Significant at the levels

*p < .05

** p < .01.

## 4. Discussion

This report provides an analysis of the housing conditions and IEQ of homes in the four First Nations communities that were part of the Sioux Lookout Zone study [24]. This work revealed the general inadequacy of the housing resulting from overcrowding, moisture damage, inadequate ventilation as well as building- and system failures.

While most of the homes were designed and built under the assumption that HRVs would be operated to provide adequate ventilation, few were actually operating. Most of the HRVs were installed in the crawlspace, thus not easily accessible for filter and core maintenance. Because The HRVs were not visible, few residents remembered to clean the filters and many units were never turned on at all. Further, cleaning outdoor intakes would often require a ladder to clean the screen on the air intake. Other problems seen during the inspections included that the installations were incomplete and not balanced. When HRVs are installed, there is a need to ensure they are balanced to limit the possibility of backdrafting from the wood stoves. There exists an opportunity to enhance awareness of the benefits of using an HRV, alongside promoting greater knowledge about operation and maintenance. Increased uptake of this technology may depend on broader education, as well as increased community-based capacity to ensure proper installation and maintenance.

The homes tended to be much smaller than the Canadian average. In 2019, the median above grade area of detached homes across Canada was 150 m^2^ [33], almost double that of homes in the present study. One notable finding was the high occupancy levels in the houses of the four communities. The average number of occupants per home was close to 7, and 45% of homes had more than one person per available room. This is a common problem in First Nations communities across Canada. Working with the 2006 Canadian Birth Census, Shapiro et al. (2021) found that overcrowding was common in First Nations with 30% of homes reporting more than one person per room [34]. In two First Nations Communities in Saskatchewan, 69% had more than one person per room [35]. For perspective, Canadian census data from 1931 to 1971 showed an average of 4 people per household, which has declined by 2.5 people over that period [36, 37]. A recent meta-analysis of overcrowding by Lorentzen et al. (2022) found that a number of conditions tend to coincide with crowding, including disrepair, biocontaminants e.g., bacteria, mold, and allergens [38].

Most of the studied homes needed minor repairs, and nearly half required major repairs. This was substantially higher than the percentages of homes in First Nations reserves needing major repairs reported in the 2006 and 2021 census data [23, 34]. While most homes did not show severe visible mold and dampness in the study, minor failures that could progress to hazardous conditions were common, similar to the findings in Elsipogtog [26]. Of critical importance, 12 homes had >1% visible mold and moisture damage per unit floor area associated with some having much greater damage. Previous studies have shown that this level of mold and moisture damage can lead to airborne spores and fragments, which can elicit a population health impact on children [19–21]. Our findings stress the importance of ventilation, which reduced visible mold, benzene and PM_2.5_ concentrations. Indoor storage of firewood and dirt floor crawlspaces were associated with glucan. Wood stoves were also associated with PM_2.5_ and benzene.

The measurement of endotoxin, fungal glucan, and allergens in settled dust provides an effective measure of exposure in occupied buildings. Room activity lofts the settled dust into the air [39]. In these communities, Kovesi et al. (2022) reported an association between endotoxin in settled dust and wheezing [24]. Similar findings have been reported by Dales et al. (2006) where airborne endotoxin was associated with infant hospitalizations in a large prospective study [40]. The geometric mean loading of endotoxin of 42368 EU /m^2^ in the present study was surprisingly high. This is much higher than that in homes in Edmonton, Wallaceburg, Windsor, Montreal, Elsipogtog or Halifax [16, 22, 26, 41].

Although the single term ‘endotoxin’ is used, the substances that make up endotoxin represent a considerable variety of different chemical structures [42–44]. Endotoxins are potent inflammatory agents when inhaled. However, the potency of endotoxin from various species of Gram-negative bacteria varies by two orders of magnitude [45]. Endotoxin chemistry in homes varies depending on the presence of potential sources such as pets, humidifiers, indoor firewood storage, and whether the building is in an urban or rural environment [46]. Endotoxin rises dramatically with the concentration of cigarette smoke in the air [47, 48]. In housing studies, Commercial tobacco smoking is a highly significant predictor of endotoxin in settled dust [44]. Little is known about the chemical forms of endotoxin in tobacco and thus their potency except they are different than from Gram negative bacteria in soil [49]. In contrast, loadings of mold glucan were lower than those of Edmonton, Halifax, Ottawa, Toronto, and Windsor homes, but higher than Montreal [12, 16, 22].

Consistent with the over-crowding, ~27% of homes had higher than desirable sustained CO_2_ concentrations of less than 1000 ppm, as suggested by Health Canada (2021) for residential settings [4]. For more than 200 years, CO_2_ has primarily been used as a proxy for ventilation which reflects many contaminants, but CO_2_ itself is not generally treated as a contaminant in homes [50]. In Nunavut, lower versus higher concentrations of CO_2_ can reduce the risk of respiratory disease in Inuit children [11]. Airborne viruses such as COVID-19 are lower in well ventilated houses [11, 51, 52]. Effective ventilation (working and maintained HRVs) is crucial for households that are crowded, with a high prevalence of commercial tobacco smoking, or wood heating to maintain good IEQ.

In summary, houses in these communities were crowded with very high concentrations of endotoxin and exposure to environmental tobacco smoke. Most of the HRVs were not functioning or used. Approximately 12% of the homes had serious mold damage resulting from both design and maintenance issues. Five decades after discussion on the quality of housing in Indigenous Communities became a public issue, there have been improvements, but many outstanding issues remain identified. A focus on the health of Indigenous children could be used to incent efforts to fund repairs and maintenance in houses.

## Supporting information

S1 Table Summary of dust mite allergen loadings from both current and previous Canadian studies, with results from older standards converted to newer standards. (XLSX)
